# Dietary Supplement With Cherry Blossom Flower and *Rosa roxburghii* Tratt Fruit Extract Improves Skin Aging: A Randomized, Placebo‐Controlled, Blinded Clinical Study

**DOI:** 10.1111/jocd.70536

**Published:** 2025-11-11

**Authors:** Daoxin Dai, Ye Di, Weihu Li, Yuhang Zhu, Siyu Chen, Baiyi Lu, Binhai Shi

**Affiliations:** ^1^ Shiseido China Innovation Center Shiseido China Co. Ltd. Shanghai China; ^2^ Key Laboratory for Quality Evaluation and Health Benefit of Agro‐Products of Ministry of Agriculture and Rural Affairs, Key Laboratory for Quality and Safety Risk Assessment of Agro‐Products Storage and Preservation of Ministry of Agriculture and Rural Affairs, College of Biosystems Engineering and Food Science Zhejiang University Hangzhou China

**Keywords:** cherry blossom flower, elasticity, *Rosa roxburghii* Tratt fruit, skin aging, skin color, wrinkle

## Abstract

**Background:**

The cherry blossom flower (sakura) and *Rosa roxburghii* Tratt (RRT) fruit demonstrate well‐established antioxidant and anti‐aging properties. However, their synergistic effects on skin aging remain understudied.

**Objective:**

This clinical study was designed to systematically evaluate the efficacy and safety of a dietary supplement containing sakura and RRT fruit extract in improving skin aging parameters.

**Methods:**

Sixty healthy female participants aged 30–50 years were randomly allocated to receive a daily dietary supplement (150 mg sakura extract + 200 mg RRT fruit extract) or a placebo for 8 weeks. Primary outcomes‐ skin hydration, chromaticity, elasticity, collagen index, texture and scaliness, and wrinkles‐ were quantitatively assessed at baseline and week 8 using standardized instrumental methods.

**Results:**

After 8 weeks of intervention, the dietary supplement significantly improved skin aging parameters versus placebo. Compared to before intervention, skin hydration increased by 28.07% (cheek) and 25.81% (forearm), significant improvement of skin chromaticity and elasticity on the cheek and inner forearm were observed (*p* < 0.01), and the mean collagen index rose by 1.8% (cheek) or 1.27% (inner forearm). Skin texture and scaliness reduction were presented. An evident reduction in the average volume and area ratio of skin wrinkles, including forehead wrinkles, crow's feet and nasolabial folds, were observed. There are reductions in neck line number (9.51%), neck line length (10.23%) and average depth of single neck line (7.14%).

**Conclusions:**

The findings indicate that the sakura and RRT fruit extract complex is a safe and effective nutraceutical for mitigating skin aging.

## 1 Introduction

Aging is an inevitable biological process characterized by progressive physiological changes in the structure, function, and appearance of the body [1, 2]. As the largest organ of the human body, skin is particularly susceptible to aging, and its associated phenotypes and rejuvenation strategies have garnered particular attention [3]. Aging skin undergoes multiple histological and morphological changes, including reduced moisture content, decreased dermal thickness, loss of elasticity, sagging, wrinkles formation, and hyperpigmentation [4, 5, 6]. These alterations are driven by a combination of intrinsic factors, such as genetics [7], ethnicity [8], hormonal fluctuation [9], as well as extrinsic factors including UV radiation [10], environmental pollution [11], and lifestyle factors like diet, habits, and stress [12].

Skin aging is primarily driven by cellular senescence [13]. Among extrinsic factors, UV irradiation, particularly the absorption of UV spectra by skin chromophores, has been extensively documented as a major contributor to skin aging phenotypes [14, 15, 16]. The key mechanism involves the UV‐induced generation of reactive oxygen species (ROS), which triggers oxidative stress in epidermal keratinocytes and dermal fibroblasts [17]. Excessive ROS accumulation can impair skin elasticity, promote pigmentation and inflammation, and cause cellular damage, collectively accelerating the aging process [18, 19]. Both intrinsic and extrinsic aging are exacerbated when oxidative stress overwhelms the skin's endogenous antioxidant defenses [20]. Also, matrix metalloproteinases (MMPs) play a critical role in the degradation of collagen, a hallmark of chronological aging [21]. Advanced Glycation End Products (AGEs) can also contribute to skin aging by inducing collagen cross‐linking, reducing skin elasticity, and promoting fibroblast apoptosis through receptor‐mediated pathways [22, 23]. These molecular changes manifest clinically as wrinkles, skin discoloration, reduced radiance, and compromised barrier function [24, 25].

Based on the understanding of biological mechanisms underlying skin aging, numerous strategies have been developed to delay its progression and promote skin health [26]. In recent years, alongside traditional skincare products and medical procedures, oral supplements have emerged as a popular approach to skin rejuvenation. Botanical extracts, particularly those rich in antioxidative flavonoids and polyphenols, are the ideal ingredients for beauty‐focused nutraceuticals, as evidenced by a growing body of clinical research. For example, a prospective clinical trial found that daily consumption of lingonberry (25 g) and amla fruit extract (30 g) for 12 weeks significantly improved stratum corneum water content, the degree of wrinkles and skin elasticity [27]. Similarly, supplementation with 250 mg of pomegranate fruit extract led to a 6.2% reduction in facial wrinkle severity after 4 weeks [28]. Furthermore, terminalia chebula supplementation (250 mg twice daily) improved facial wrinkles by 4.3% over 8 weeks in women aged 25–65 [29].

The cherry blossom flower (sakura), a member of the Rosaceae family, is abundant in bioactive compounds such as flavonoids, polyphenols, triterpenes, and polysaccharides. It has a long‐standing history of use in traditional Chinese medicine and cuisine [30, 31]. Sakura is known for its diverse biological properties, including anti‐glycation, antioxidant, anti‐inflammatory, and anti‐cancer effects [32, 33, 34, 35]. Specifically, Sakura extracts contain cinnamoyl glucose derivatives and flavonol glucosides, which have been proven to effectively reduce AGEs [32] and inhibit UV‐induced matrix metalloproteinase‐1 (MMP‐1) expression through the MEK1/2‐ERK pathway, presenting promise as an anti‐wrinkle and photoaging preventive agent [36]. Furthermore, studies have shown that chewable tablets containing lingonberry and sakura extracts have notable anti‐glycation effects [37]. In addition to sakura, research indicates that RRT extract boosts telomerase activity, promoting DNA replication and telomere maintenance pathways, thereby exhibiting potential as a natural anti‐aging agent [38].

Previous large‐scale in‐house in vivo screening tests have found that compounds containing RRT fruit and sakura extract can effectively boost collagen I synthesis and show significant synergistic efficacy (unpublished data). Despite these promising findings, the combined effects of RRT fruit and sakura extracts on skin aging have not been thoroughly investigated in clinical trials. Therefore, this study aimed to evaluate the skin aging efficacy of a dietary supplement containing RRT fruit and sakura extracts after prolonged consumption, addressing a critical gap in the current literature.

## 2 Materials and Methods

### 2.1 Study Design and Ethical Aspects

The study was a randomized, placebo‐controlled, single‐blind clinical trial conducted at the Skin Research Center of Landproof Testing Technology Co. Ltd. Ethical approval was granted by the Ethics Committee of Guangdong Daily Chemical Industry (Approval No. GDIRB [2024]4‐3, approved date: April 30, 2024), and all participants provided written informed consent. Participants were randomly assigned to the experimental group or placebo group using a computer‐generated sequence of 62 nonunique, unsorted numbers with a range from 1 to 2 representing the groups. The principal investigator kept the allocation sequence confidential and performed group assignments.

### 2.2 Study Participants

A total of 62 healthy female participants aged 30 to 50 years were enrolled in this study. The average age of experimental and placebo group was 45.13 ± 3.44 and 45.83 ± 2.72 years, respectively. All participants exhibited mild signs of skin aging, including dryness, dull complexion, and visible wrinkles such as forehead lines, crow's feet, and neck lines. Eligibility for the study was confirmed by a dermatologist through physical examination.

Inclusion criteria were as follows: (1) healthy skin without chronic conditions, (2) ability to understand and comply with the study protocol, and (3) willingness to avoid other skincare products or treatments during the study period.

Exclusion criteria included the following: (1) use of products containing active ingredients within 1 month before the study, (2) visible skin conditions such as sunburn or hyperpigmentation, (3) history of chronic skin diseases or cosmetic procedures, and (4) any condition likely to interfere with study outcomes.

Informed consent was obtained from all participants before enrollment.

Two participants (one per group) were excluded due to non‐responsiveness, resulting in 60 cases for final analysis.

### 2.3 Study Schedule

**FIGURE 1:**
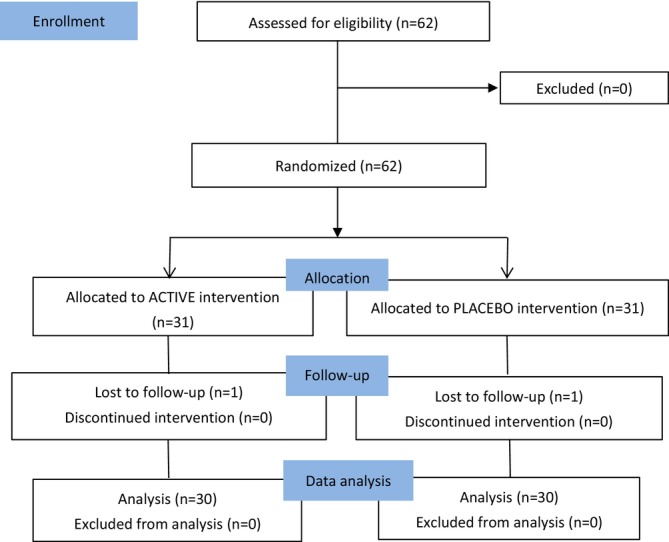
Participants' flow diagram.

The study duration was 8 weeks, and physiological parameters of skin aging were measured before the first consumption of the experimental product (D0) and after 56 days of consumption (D56). Efficacy data were documented throughout the trial process (Figure 1). All participants demonstrated normal medical examination results, including vital signs (blood pressure, heart rate), hematological parameters (RBC, WBC, hemoglobin, platelet count), blood biochemistry (total protein, albumin, bilirubin, AST, ALT, BUN, creatinine, uric acid, total cholesterol, triglycerides), and urinalysis (color clarity, specific gravity, pH, protein, glucose, blood, nitrite), before and after administration of the experimental product. Meanwhile, the safety and tolerability of the product were monitored by dermatologists through dermatological examinations, interviews and daily adverse event questionnaire documenting: gastrointestinal symptoms (nausea, vomiting, abdominal pain, bloating, diarrhea, constipation, appetite changes), neurological symptoms (dizziness, headaches, insomnia), dermatological manifestations (erythema, desquamation, rash), and cardiovascular symptoms (palpitations, chest tightness). Based on physician assessments and the adverse event questionnaire, no adverse events were recorded throughout the study duration.

### 2.4 The Experimental Product and Placebo Product

The experimental group received a powder sachet with 150 mg sakura extract and 200 mg RRT fruit extract. This ratio showed the best cellular anti‐aging effect in in‐house tests (unpublished data). The active substance in the placebo powder sachet was replaced by maltodextrin. In addition, all samples shared the same excipients composition, including erythritol, mogrosides, grapefruit and jasmine flavoring essence.

The dried “Kanzan” sakura flowers were extracted with water (1:20, w/w) at 50°C for 1 h. The resulting solution was filtered through a 200 nm ultrafiltration membrane, followed by reverse osmosis concentration to 10% solid content. Then the concentrated solution was mixed with 50% maltodextrin and lyophilized to obtain a sakura extract. Similar processing methods were also used in RRT fruit extract preparation. Instead, filtered RRT fruit juice was concentrated to 15% solid content using nanofiltration, followed by further vacuum concentration at 60°C. Finally, 30% Maltodextrin was added, and the mixture was spray‐dried to obtain the RRT fruit extract.

Participants were asked to directly take experimental or placebo powder sachet once daily for 56 days. Compliance was monitored weekly, and participants were provided with detailed usage instructions to ensure adherence to the intervention protocol.

### 2.5 Efficacy Measurements

Efficacy measurements were conducted at baseline (D0) and post‐intervention (D56) to evaluate the effects of the experimental product on various skin parameters. All assessments were performed under standardized conditions, with participants acclimating for 30 min in a controlled environment (temperature: 21°C ± 1°C, relative humidity: 50% ± 5%) before measurements.

#### 2.5.1 Skin Hydration

Skin hydration was assessed using the Corneometer CM825 (Courage and Khazaka electronic GmbH, Germany) on the cheek and inner forearm. Three measurements were taken at each site, and the average value was used for analysis.

#### 2.5.2 Skin Chromaticity

Skin chromaticity parameters, including L* (brightness), b* (yellowness), and melanin content, were measured on the cheek and inner forearm using the spectrophotometer (CM‐26d, Konica Minolta, Japan) and Mexameter MX18 (Courage and Khazaka electronic GmbH, Germany). ITA° was calculated using the formula ITA° = [arctan (L* − 50)/b*] × 180°.

#### 2.5.3 Skin Elasticity

Skin elasticity was evaluated on the cheek and inner forearm using the Cutometer Dual MPA 580 (Courage and Khazaka electronic GmbH, Germany), which measured five key parameters: R0 (softness), R2 (total elasticity), R5 (net elasticity), R7 (elastic recovery), and F4 (firmness). Higher R0, R2, R5, and R7 values indicate improved elasticity, while lower F4 values reflect better firmness.

#### 2.5.4 Collagen Index

The collagen index was assessed on the cheek and inner forearm using the SIAscope (Astron Clinica, Cambridge, UK), a portable scanning device that quantifies collagen content.

#### 2.5.5 Skin Texture and Skin Scaliness

Skin texture and scaliness were evaluated on the outer calf using the Visioscan VC 20plus (Courage and Khazaka Electronic GmbH, Germany). Key parameters included SEw (skin texture) and SEsc (scaliness degree), with lower values indicating improved skin smoothness and reduced scaliness.

#### 2.5.6 Skin Wrinkles

Wrinkles on the forehead, crow's feet, and nasolabial folds were assessed using the PRIMOS CR system (Canfield Co. USA) to capture 3D topographic images, with wrinkle volume analyzed using dedicated software.

The Visia‐CR system (Canfield Imaging Systems, Fairfield, NJ, USA) was used to obtain facial photographs, and Image‐Pro‐Plus software (Media Cybernetics Co. USA) calculated wrinkle area ratios for further evaluation.

The Antera 3D scanner (Miravex Limited, Dublin, Ireland) measured the average depth, number, and length of neck lines, as well as average depth of single crow's feet, to provide a comprehensive assessment of wrinkle severity.

### 2.6 Statistical Analysis

All statistical analyses were performed using SPSS 21.0 software. Intra‐group comparisons between baseline (D0) and post‐intervention (D56) measurements were conducted using the paired sample *t*‐test for normally distributed data or the Wilcoxon signed‐rank test for non‐parametric data. Moreover, an independent samples *t*‐test or Wilcoxon rank‐sum test was applied to examine differences in the relative values of test parameters (D56–D0) between the experimental product and the placebo group. A two‐tailed test was used for data analysis with a significance level *α* = 0.05.

## 3 Results

### 3.1 Skin Hydration Improvement

Higher skin hydration indicated increased water content within the skin's stratum corneum. Skin hydration significantly increased after 8 weeks of intervention (cheek: +28.07% vs. D0, *p* < 0.001; inner forearms: +25.81% vs. D0, *p* < 0.001). In contrast, no significant changes were observed in the placebo group at D56 for either the cheek or inner forearm (cheek: +2.62% vs. D0, *p* = 0.520; inner forearms: +0.88% vs. D0, *p* = 0.524). The relative changes in skin hydration (D56–D0) were statistically significant (*p* < 0.001) between groups in favor of the experimental product.

### 3.2 Skin Chromaticity Improvement

**FIGURE 2:**
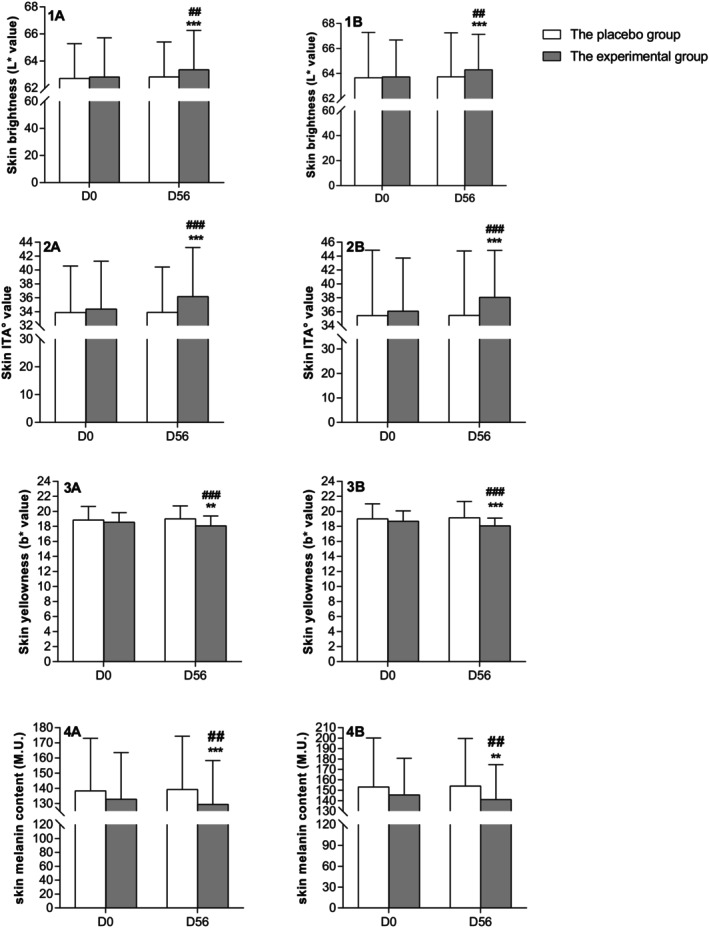
Skin chromaticity parameters improvement after 56 days of intervention. ** and *** denote *p* < 0.01 or *p* < 0.001 versus baseline, respectively; ## and ### denote *p* < 0.01 and *p* < 0.001 versus the placebo group, respectively. 1A, 2A, 3A, and 4A indicated the results of the cheek, and 1B, 2B, 3B, and 4B showed the results of the inner forearm.

**FIGURE 3:**
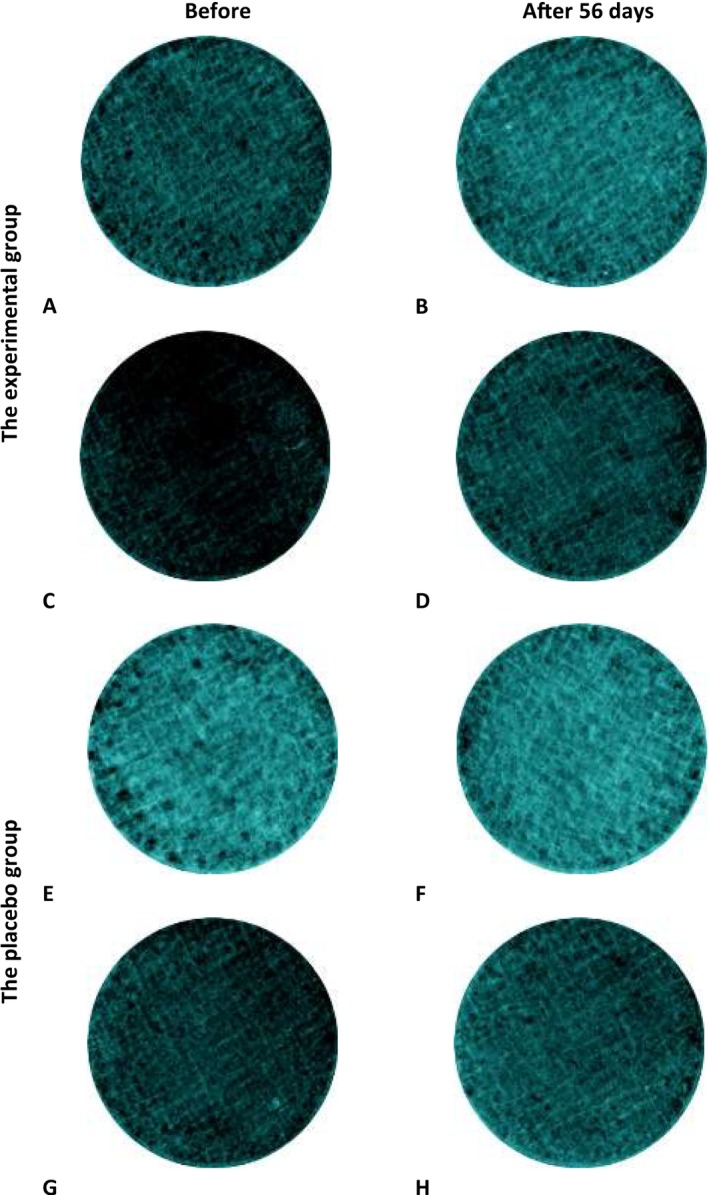
Representative images before and after 8 weeks using SIAscope. Photographs (A), (B), (E), and (F) represent inner forearm condition. Photographs (C), (D), (G), and (H) represent cheek condition.

Significant improvements have been observed in the skin chromaticity parameters after 56 days of intervention. In the experimental group, skin brightness (L* value) and skin ITA° on the cheek were increased by 0.89% and 5.27%, respectively (*p* < 0.001), and the same trend was present on the inner forearm (*p* < 0.001) (Figure 2–1A,B,2A,B); whereas yellowness (b* value) and melanin content are significantly reduced either cheek or inner forearm (Figure 2–3A,B,4A,B). No significant difference was found for skin chromaticity parameters measured in this study following the placebo intake. The differences between groups were proven to be statistically significant.

### 3.3 Skin Elasticity Enhancement

Skin elasticity is an important indicator of skin aging, assessed through five key parameters. Following a 56‐day supplementation period, significant improvements in elasticity parameters (R0, R2, R5, R7, and F4) were observed, while no significant differences were noted in the placebo group, both on the cheek and inner forearm (Table 1).

**TABLE 1:** Skin elasticity enhancement on the cheek and inner forearm.

Parameter	Group	Cheek				Inner forearm			
		D0	D56	Changing rate (%)	*p* (inter‐group)	D0	D56	Changing rate (%)	*p* (inter‐group)
R0	EP	0.311 ± 0.073	0.332 ± 0.071***	↑6.58	< 0.001	0.358 ± 0.059	0.377 ± 0.061***	↑5.29	< 0.001
	PP	0.314 ± 0.073	0.311 ± 0.066	↓0.88		0.372 ± 0.078	0.369 ± 0.070	↓0.72	
R2	EP	0.662 ± 0.061	0.696 ± 0.048***	↑5.12	< 0.001	0.771 ± 0.035	0.801 ± 0.036***	↑3.85	< 0.001
	CP	0.643 ± 0.061	0.642 ± 0.060	↓0.14		0.776 ± 0.039	0.772 ± 0.040	↓0.53	
R5	EP	0.548 ± 0.059	0.578 ± 0.060***	↑5.33	< 0.001	0.693 ± 0.118	0.722 ± 0.104***	↑4.17	< 0.001
	CP	0.521 ± 0.072	0.518 ± 0.069	↓0.47		0.721 ± 0.138	0.712 ± 0.145	↓1.18	
R7	EP	0.417 ± 0.055	0.466 ± 0.058***	↑11.98	< 0.001	0.540 ± 0.102	0.580 ± 0.092***	↑7.38	< 0.001
	CP	0.401 ± 0.067	0.399 ± 0.072	↓0.58		0.578 ± 0.132	0.571 ± 0.128	↓1.31	
F4	EP	16.04 ± 2.40	14.80 ± 1.86***	↓7.70	0.008	17.51 ± 2.08	15.47 ± 1.55***	↓11.70	< 0.001
	CP	16.09 ± 2.47	16.23 ± 2.56	↑0.86		18.49 ± 2.71	18.60 ± 2.68	↑0.55	

*Note:* Data are expressed as mean ± SD. Significant differences were observed between the baseline (D0) and after 56 days of intervention with *p* ≤ 0.001 (***). Change value from baseline was analyzed, and results are presented as *p* value (inter‐group).

Abbreviations: EP, experimental product; PP, placebo product.

### 3.4 Mean Collagen Index Improvement

The SIAscope could be used to evaluate how the metabolism of key molecules in the skin, such as collagen, is affected by oral supplements. An elevated mean collagen index suggests a higher collagen content within the skin. The mean collagen index was significantly increasing (cheek: +1.82% vs. D0; inner forearms: +1.27% vs. D0) at D56 in experimental group (*p* < 0.001), and showed no significant difference in the placebo group compared to baseline, and Figure 3 showed representative images captured before and after 8‐week ingestion using SIAscope, Meanwhile, there was a statistical significance of skin collagen relative change value (D56–D0) between two groups (*p* < 0.05).

### 3.5 Skin Texture and Scaliness Degree Reduction

**FIGURE 4:**
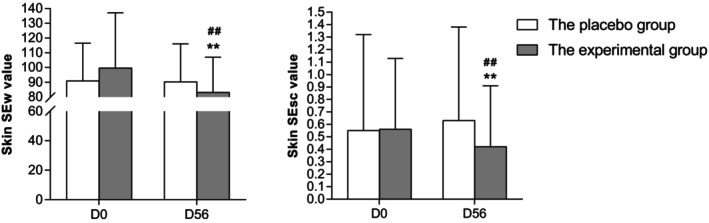
Skin wrinkle (SEw value) and skin scaliness degree (SEsc value) reduction on the outer calf. ** denotes *p* < 0.01 versus baseline; ## denotes *p* < 0.01 versus the placebo group.

Figure 4 shows the descriptive analysis of skin SEw and SEsc before intake and after 8 weeks of intake. The SEw and SEsc values decreased significantly by 16.54% and 26.15% in the experimental group, respectively. The improvement degree of two parameters (D56–D0) with the experimental product was superior to the placebo (*p* < 0.01).

### 3.6 Skin Wrinkle Improvement in the Experimental Group

**FIGURE 5:**
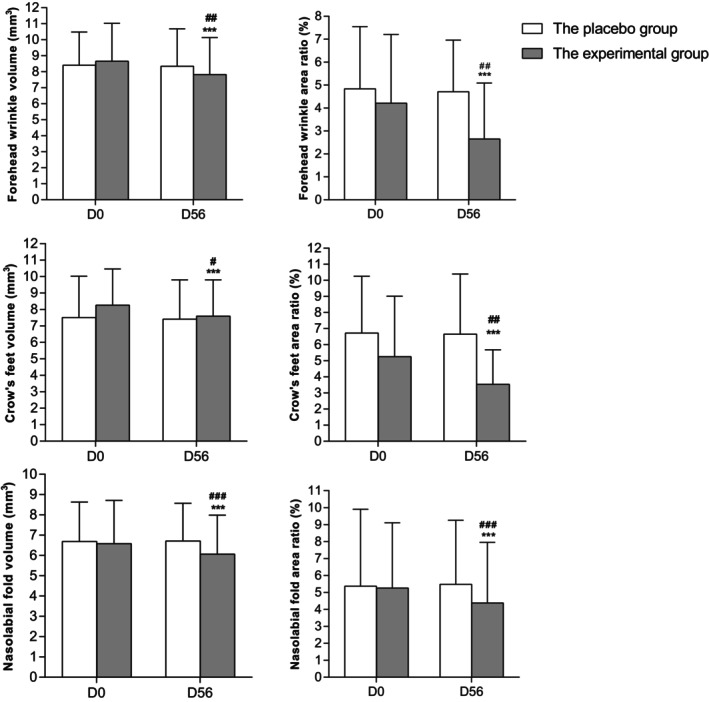
Forehead wrinkles, crow's feet and nasolabial folds improvement. *** denotes *p* < 0.001 versus baseline; #, ##, and ### denote *p* < 0.05, *p* < 0.01, and *p* < 0.001 versus the placebo group.

**FIGURE 6:**
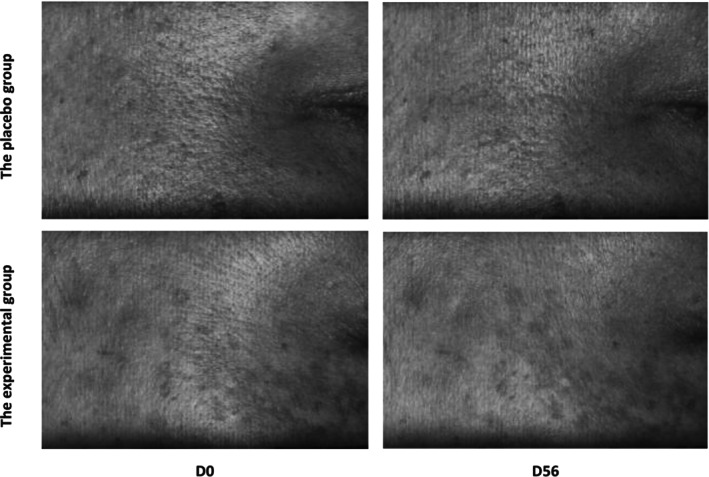
Representative improving images on crow's feet using PRIMOS CR.

The data analysis showed an evident reduction in the average volume of skin wrinkle including forehead wrinkles, crow's feet and nasolabial folds, after 8 weeks of intake (forehead wrinkle: −9.62%; crow's feet: −8.23%; nasolabial folds: −7.93%) (*p* < 0.001), the variation of all relative parameters (D56–D0) between two groups was statistically significant (forehead wrinkles: *p* < 0.01; crow's feet: *p* < 0.05; nasolabial folds: *p* < 0.001). The wrinkle area in these three regions had a similar trend, the results are depicted in Figure 5. Representative improving images on crow's feet are presented in Figure 6.

A statistically significant decrease in the average depth of single crow's feet was observed in the treatment group (*p* < 0.001), and this reduction was greater in the experimental group than the placebo group (−12.37% vs. −1.21%).

**FIGURE 7:**
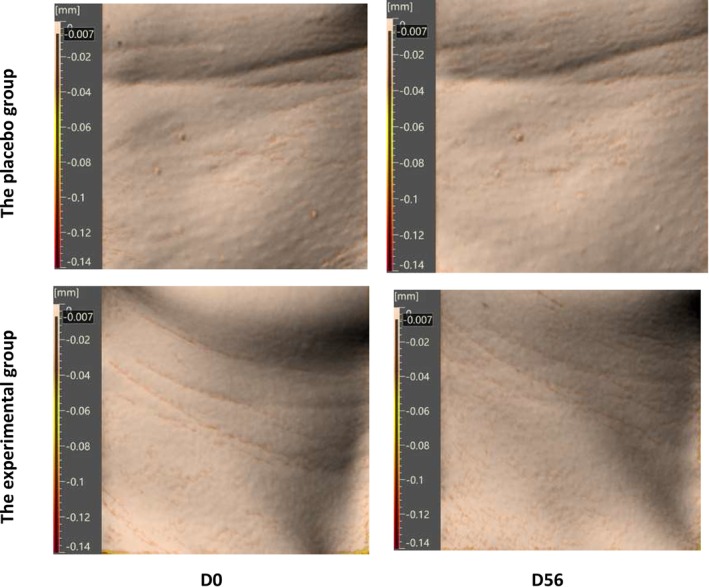
Neck line improvement observed using Antera 3D.

Furthermore, improvement efficacy on neck lines was assessed through Antera 3D, and the results showed a reduction in neck line number (−9.51%), neck line length (−10.23%) and average depth of single neck line (−7.14%) at D56, and greater improvements were observed in the experimental group compared to the placebo group (Table 2). Representative images obtained from Antera 3D are reported in Figure 7.

**TABLE 2:** The analysis results of the neck lines after ingestion of the test product.

Parameter	Group	D0	D56	Changing rate (%)	*p* (inter‐group)
Neck line number	EP	70.10 ± 24.89	63.43 ± 23.46***	↓9.51	< 0.001
	PP	81.13 ± 25.24	83.87 ± 26.45	↑3.37	
Neck line length (mm)	EP	408.31 ± 156.76	366.55 ± 144.43***	↓10.23	< 0.001
	PP	493.47 ± 181.62	499.78 ± 186.95	↑1.28	
Average depth of single neck line (mm)	EP	0.01485 ± 0.00179	0.01379 ± 0.00162***	↓7.14	0.005
	PP	0.01600 ± 0.00293	0.01602 ± 0.00226	↑0.10	

*Note:* Data are expressed as mean ± standard deviation. Significant differences between the baseline (D0) and the follow‐up time (D56) (***p ≤ 0.001). Change value from baseline was analyzed and results are presented as *p* value (Inter‐group).

Abbreviations: EP, experimental product; PP, placebo product.

## 4 Discussion

Antioxidant‐enriched plant extracts have garnered increasing attention in recent years due to their natural and safe properties, making them popular functional ingredients in dietary supplements aimed at combating skin aging [39]. This randomized, placebo‐controlled clinical trial is the first to demonstrate the anti‐aging effects of the combination of sakura and RRT fruit. In Chinese women aged 30–50, this consumption could significantly improve multiple signs of skin aging, including increased skin hydration, elasticity enhancement, higher collagen index, skin color improvement and wrinkle fading. Notably, positive results were observed in nearly all participants, underscoring the broad efficacy of the intervention.

The skin's connective tissue, primarily composed of collagen and elastin, plays a critical role in maintaining skin structure and elasticity. Collagen provides firmness and tensile strength, while elastin allows the skin to stretch and rebound [40]. With aging, the degradation of these proteins leads to sagging skin, fine lines, and deep wrinkles [41, 42]. In vitro studies have shown that sakura extract protects HaCaT cells from UVB‐induced oxidative stress and apoptosis by decreasing the levels of ROS and malondialdehyde, while increasing the activities of antioxidant enzymes such as superoxide dismutase and glutathione peroxidase [43]. Our previous research also found that the combination of sakura and RRT extract promoted the synthesis of elastin and collagen in dermal fibroblasts at optimal concentrations (internal unpublished data). In the current study, this combination demonstrated significant improvements in skin elasticity parameters (R0, R2, R5, R7), which were further confirmed by an increase in the average collagen index. By mitigating the degradation of collagen and elastin, which are essential for maintaining skin firmness, the product effectively combats the loss of skin elasticity and the formation of wrinkles. The earlier study found that intake of cherry blossom extract alone (150 mg/day for 8 weeks) had no effect on skin elasticity [44], the combination with RRT fruit in this study yielded significant improvements, likely due to the enhanced antioxidant properties of the experimental product.

Wrinkles, a prominent sign of skin aging, are largely driven by oxidative stress and ROS [20]. Although earlier studies reported that the 8‐week ingestion of standardized sakura extract did not lead to improvements in wrinkles based on instrument measurements. However, subjective evaluation using Visual Analog Scale (VAS) scores for “wrinkles around the eyes” remained stable in the group receiving the extract but deteriorated in the placebo group [44]. In this trial, it was discovered that the combination of sakura and RTT fruit extract could extensively improve facial wrinkles, including crow's feet, forehead wrinkles, and nasolabial folds, as evidenced by reductions in volume and area ratios. There is a noticeable enhancement in the average depth of the crow's feet. More importantly, the study demonstrates for the first time the anti‐aging efficacy of sakura extract and RRT fruit on the neck fines. Similar to facial skin, neck skin aging is influenced by both intrinsic and extrinsic factors that promote loss of skin elasticity and further wrinkle formation, resulting from the gradual degradation of collagen and elastin [45]. However, the neck skin was found to be more extensible, elastic, and viscoelastic than the cheek. Additionally, the dermal layer of the neck skin was thinner but more intense than that of the cheek [46]. The present study substantiated that the neck line became less visible after 56 days of product ingestion. Nowadays, the number of people with neck lines has increased due to the tendency of many people to tilt their heads down when using a computer or smartphone. The test product provides an effective solution to this problem. This suggests that the combination was more effective than sakura extract alone in enhancing skin elasticity and reducing wrinkles. Moreover, Skin texture (SEw) and scaliness degree (SEsc) in our study showed significant improvements after 8 weeks of consumption, aligning with the results observed for skin elasticity and moisture.

Secondly, skin hydration, as the basic foundation of healthy skin, is essential for maintaining proper skin condition, which is directly related to skin smoothness. As individuals age, a natural decline in skin hydration occurs. Previous research found that the ingestion of sakura extract (150 mg/day) for 8 weeks did not affect skin moisture [44], however, the present study demonstrated that the consumption of a combination of sakura and RRT extract resulted in significant improvement to skin moisture. This suggests that the synergistic effect of sakura and RRT extract on skin moisture and elasticity may be more potent than sakura extract alone. On the other hand, concerning the effectiveness between the cheek and the inner forearm, it was noted that cheek moisture exhibited slightly better improvements compared to the inner forearm. The phenomenon may be attributed to the structural and tissue differences between facial skin and inner forearm skin [47].

Skin whitening and brightening is one of the priority needs for individuals of all ages and genders. Melanin overproduction, driven by cell degradation and UV exposure, leads to facial spots and uneven skin tone [48]. Sakura extract has been found to reduce skin AGEs and reduce facial pigmentation and reddish after an 8‐week ingestion period [42]. While no human efficacy studies have been reported for RRT fruit, its functional constituents—such as polysaccharides, vitamin C, superoxide dismutase (SOD), and phenolic compounds—are known for their antioxidant properties, which combat oxidative stress and aging [31]. a novel water‐soluble polysaccharide (RRTP1‐1) obtained from RRT fruit showed effective antioxidant activity in vitro and in vivo and could be potential antioxidants for functional food [49]. In this study, the combination significantly brightened facial and forearm skin, reduced melanin levels, and improved skin yellowness. This aligns with findings that oral supplementation with antioxidant‐rich compounds, such as melon concentrate enriched in SOD, can enhance endogenous antioxidant enzymes and reduce melanin levels [50].

In contrast, the placebo group showed no significant changes in skin moisture, elasticity, tone, or wrinkles. The degree of improvement in the experimental group exceeded that of the placebo group across all parameters. Unlike many similar studies that evaluate a limited number of skin aging parameters [51, 52], our study stands out by comprehensively examining almost all relevant aspects of skin aging. By addressing a wide array of parameters, we aim to provide a more holistic understanding of the effects and potential benefits associated with our investigational product.

To explore the anti‐aging effects of the product comprehensively, efficacy measurements are not restricted to the facial region but extended to areas including the arms and neck. This inclusive approach provides a broader comprehension of the anti‐aging effectiveness of the experimental product.

## 5 Conclusion

This study demonstrated that dietary supplements containing 150 mg sakura and 200 mg RRT extract complex can comprehensively improve facial and body skin aging phenotypes compared to placebo. Specifically, skin moisture on cheek and forearm significantly increased 28.07% and 25.81%, respectively. Related skin roughness in‐dex also showed 26.15% positive improvement after intaking. For holistic anti‐wrinkle efficacy, the area of forehead wrinkles, crow's feet and nasolabial folds significantly decreased 37.07%, 32.78%, and 16.76%, respectively. Similarly, significant reduction of wrinkles depth and volume were also observed in experimental group. And the neck line number, length and average depth of single line were reduced 9.51%, 10.23%, and 7.14%. As for skin tone improvement, the brightness index increased (cheek 0.86%, forearm 0.91%), and yellowness parameter decreased significantly (cheek 2.06%, forearm 3.31%). For skin elasticity, all indexes (R0, R2, R5, R7) in experimental group showed significant improvement, which is in line with detected skin collagen content increased by SIAscope (cheek 1.82%, forearm 1.27%). Oral supplements with botanical extracts, as the new approach of skin anti‐aging, represent a scientifically supported and viable alternative to topical treatments for individuals seeking comprehensive anti‐aging benefits.

## Author Contributions

D.D., Y.D., and B.S. designed research; D.D. and Y.D. wrote the manuscript; D.D., W.L., Y.Z., and S.C. performed research; D.D., Y.D., B.L., and B.S. reviewed and revised the manuscript. All authors contributed to the article and approved the submitted version.

## Ethics Statement

This study was conducted in compliance with the declaration of Helsinki and was also approved by the Ethics Committee of Guangdong Daily Chemical Industry (Approval No. GDIRB [2024]4‐3; Approved date: April 30, 2024) prior to the initiation of the study, and informed consent was obtained from each participant before any procedures were performed.

## Conflicts of Interest

The authors declare no conflicts of interest.

## Data Availability

The data that support the findings of this study are available on request from the corresponding author. The data are not publicly available due to privacy or ethical restrictions.
